# Supplementary material for: Modeling glioblastoma heterogeneity as a dynamic network of cell states
Source: Mol Syst Biol. 2021 Sep 16;17(9):e10105. doi: 10.15252/msb.202010105 (PMC8444284; doi:10.15252/msb.202010105)
Supplement: Supplementary file 5 — Source Data for Figure 3 [file MSB-17-e10105-s001.zip › Figure3A_sourcedata/GSEA_3065/nosigdb_state4.GseaPreranked.1622718430994/SUVA_G1S_PMID_31327527.html]

Details for gene set SUVA\_G1S\_PMID\_31327527[GSEA]

|  || Dataset | state4 |
| Phenotype | NoPhenotypeAvailable |
| Upregulated in class | na\_neg |
| GeneSet | SUVA\_G1S\_PMID\_31327527 |
| Enrichment Score (ES) | -0.9165029 |
| Normalized Enrichment Score (NES) | -2.6059363 |
| Nominal p-value | 0.0 |
| FDR q-value | 0.0 |
| FWER p-Value | 0.0 |
Table: GSEA Results Summary

  

Fig 1: Enrichment plot: SUVA\_G1S\_PMID\_31327527      
 Profile of the Running ES Score & Positions of GeneSet Members on the Rank Ordered List

  

| PROBE | GENE SYMBOL | GENE\_TITLE | RANK IN GENE LIST | RANK METRIC SCORE | RUNNING ES | CORE ENRICHMENT || 1 | RNASEH2A |  |  | 5241 | -0.105 | -0.8294 | No |
| 2 | MCM2 |  |  | 5785 | -0.180 | -0.8978 | Yes |
| 3 | CCNE2 |  |  | 5827 | -0.190 | -0.8847 | Yes |
| 4 | PKMYT1 |  |  | 5959 | -0.225 | -0.8824 | Yes |
| 5 | TMEM106C |  |  | 5979 | -0.230 | -0.8616 | Yes |
| 6 | FEN1 |  |  | 6035 | -0.256 | -0.8439 | Yes |
| 7 | CDC45 |  |  | 6063 | -0.276 | -0.8196 | Yes |
| 8 | GMNN |  |  | 6080 | -0.285 | -0.7926 | Yes |
| 9 | CDCA5 |  |  | 6099 | -0.296 | -0.7649 | Yes |
| 10 | DUT |  |  | 6107 | -0.303 | -0.7346 | Yes |
| 11 | MCM3 |  |  | 6113 | -0.307 | -0.7036 | Yes |
| 12 | MELK |  |  | 6117 | -0.312 | -0.6718 | Yes |
| 13 | ASF1B |  |  | 6127 | -0.320 | -0.6401 | Yes |
| 14 | AURKB |  |  | 6143 | -0.332 | -0.6080 | Yes |
| 15 | TK1 |  |  | 6144 | -0.334 | -0.5734 | Yes |
| 16 | RRM1 |  |  | 6147 | -0.338 | -0.5387 | Yes |
| 17 | RAD51AP1 |  |  | 6167 | -0.357 | -0.5048 | Yes |
| 18 | CENPK |  |  | 6169 | -0.363 | -0.4673 | Yes |
| 19 | MCM4 |  |  | 6171 | -0.372 | -0.4289 | Yes |
| 20 | PCNA |  |  | 6173 | -0.374 | -0.3903 | Yes |
| 21 | CENPM |  |  | 6179 | -0.386 | -0.3511 | Yes |
| 22 | GINS2 |  |  | 6194 | -0.420 | -0.3099 | Yes |
| 23 | ZWINT |  |  | 6195 | -0.425 | -0.2659 | Yes |
| 24 | RRM2 |  |  | 6199 | -0.438 | -0.2210 | Yes |
| 25 | CKS1B |  |  | 6234 | -0.523 | -0.1722 | Yes |
| 26 | MCM7 |  |  | 6252 | -0.665 | -0.1060 | Yes |
| 27 | HIST1H4C |  |  | 6262 | -1.038 | 0.0002 | Yes |
Table: GSEA details [plain text format]

  

Fig 2: SUVA\_G1S\_PMID\_31327527: Random ES distribution      
 Gene set null distribution of ES for **SUVA\_G1S\_PMID\_31327527**
